# Severe enterovirus A71 associated hand, foot and mouth disease, Vietnam, 2018: preliminary report of an impending outbreak

**DOI:** 10.2807/1560-7917.ES.2018.23.46.1800590

**Published:** 2018-11-15

**Authors:** Le Nguyen Thanh Nhan, Nguyen Thi Thu Hong, Le Nguyen Truc Nhu, Lam Anh Nguyet, Nguyen Thi Han Ny, Tran Tan Thanh, Do Duong Kim Han, Hoang Minh Tu Van, C Louise Thwaites, Tran Tinh Hien, Phan Tu Qui, Pham Van Quang, Ngo Ngoc Quang Minh, H. Rogier van Doorn, Truong Huu Khanh, Nguyen Van Vinh Chau, Guy Thwaites, Nguyen Thanh Hung, Le Van Tan

**Affiliations:** 1 Children’s Hospital 1, Ho Chi Minh City, Vietnam; 2 Oxford University Clinical Research Unit, Ho Chi Minh City, Vietnam; 3 Centre for Tropical Medicine and Global Health, Nuffield Department of Medicine, University of Oxford, Oxford, United Kingdom; 4 Hospital for Tropical Diseases, Ho Chi Minh City, Vietnam

**Keywords:** Enteroviruses, enterovirus A71, hand, foot and mouth disease, Vietnam

## Abstract

Since January 2018, over 53,000 hospitalisations and six deaths due to hand, foot and mouth disease (HFMD) have occurred across Vietnam with most cases from September onward. In a large tertiary referral hospital, Ho Chi Minh City, enterovirus A71 subgenogroup C4 was predominant, while B5 was only sporadically detected. The re-emergence of C4 after causing a severe HFMD outbreak with > 200 deaths in 2011–12 among susceptible young children raises concern of another impending severe outbreak.

Since the beginning of January 2018, there has been a significant increase in the number of hospitalisations (including cases with neurological symptoms) due to hand, foot and mouth disease (HFMD) across Vietnam. By the end of September more than 53,000 clinical cases were reported, of whom six died [1]. Our aim was to characterise the epidemiology, virology and clinical characteristics of patients, especially those with severe HFMD, through an ongoing clinical study conducted at a large tertiary referral hospital for children in Ho Chi Minh City, Vietnam from January to September 2018.

## Setting and outbreak investigations

Children’s Hospital 1 (CH1) in Ho Chi Minh City, Vietnam is a 1,600-bed hospital, and is one of the three tertiary referral centres for children with HFMD in Ho Chi Minh City. The hospital admits children from southern Vietnam with a catchment population of over 40 million. 

According to the Vietnamese Ministry of Health, HFMD is clinically divided into four major grades: Grade 1 is assigned to patients presenting with mouth ulcers or vesicles/papules on hands, feet or buttocks, with or without mild fever (< 39 °C); Grade 2 is further divided into Grade 2a (central nervous system (CNS) involvement, (myoclonus reported by parents or caregivers only, fever > 39 °C or ataxia)), Grade 2b1 (myoclonus observed by medical staff or history of myoclonus and lethargy or pulse higher than 130 bpm), and Grade 2b2 (ataxia, cranial nerve palsies, limb weakness, nystagmus, persistent high fever or pulse higher than 150 bpm); Grade 3 involves autonomic dysfunction with sweating, hypertension, tachycardia and tachypnoea and Grade 4 is for disease with additional cardio-pulmonary compromise with pulmonary oedema or shock syndrome [2]. 

The data presented here were either derived from the hospital database record or obtained from cases enrolled into an ongoing clinical study conducted at CH1 [3]. Since January 2018, our recruitment focus has been inpatients, especially those with severe HFMD, with a targeted ratio of 1:1 for Grade 2a and 2b1 vs 2b2 or above. 

Recruited patients had throat and rectal swabs collected on admission alongside clinical data during hospitalisation. The study was approved by the Institutional Review Board of CH1 and the Oxford Tropical Research Ethics Committee (OxTREC).

Multiplex PCR for simultaneous detection of enteroviruses (EVs) and enterovirus A71 (EV-A71) was performed on rectal and throat swabs [4]. PCR positive swabs were then subjected to viral protein 1 (VP1) nucleotide sequencing using previously described PCR primers [2,5,6]. The obtained PCR amplicons were sequenced in both directions using corresponding PCR primers and BigDye Terminator V3.1 Cycle Sequencing kit in an ABI 3130XL DNA sequencer (Applied Biosystems, Carlsbad, CA, United States (US)). EV-A71 subgenogroup determination and EV serotyping were then carried out using the obtained VP1 sequences and an online EV typing tool [7].

### Description of the epidemic and characteristics of the patients 

**Figure 1:**
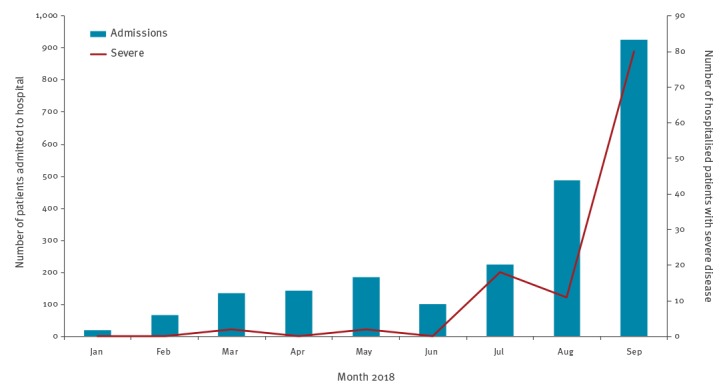
Monthly distribution of HFMD cases and HFMD cases with severe disease admitted to Children’s Hospital 1, Vietnam, January–September 2018 (n = 2,283)

CH1 recorded 2,283 hospitalisations due to HFMD since January 2018, of whom, 113 (5%) had severe HFMD (Grade 2b1, 2b2, 3 or 4) (Figure 1). A total of 1,376 (60%) hospitalisations concerned boys, and 2,250 (99%) were in children ≤5 years old. The number of admissions (n = 1,411) and cases with severe disease (n = 91) in August and September combined was 1.6 and 4.1 times, respectively, that of the first 7 months of the year combined (Figure 1). 

HFMD: hand, foot and mouth disease.

**Figure 2:**
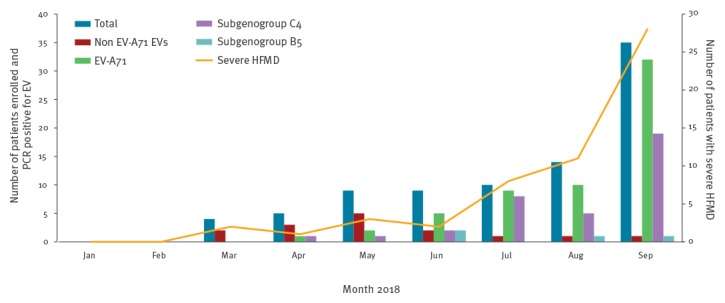
Monthly distribution of clinical HFMD cases, including those who were PCR positive for EV-A71 or non-EV-A71 EVs, and clinical cases with severe HFMD enrolled in the study, Vietnam, January–September 2018 (n = 86 patients)

EV: enterovirus; HFMD: hand, foot and mouth disease.

Of 86 clinically diagnosed HFMD patients enrolled in the clinical study, 55 (64%) had severe HFMD, 54 (63%) were boys, and 85 (99%) were ≤ 5 years old. As for CH1, the number of cases with neurological symptoms and/or autonomic dysfunction enrolled in our study sharply increased compared with previous months, in August (n = 11) and September (n = 28), accounting for 39/55 (71%) of all cases enrolled with severe HFMD in 2018 (Figure 2). One patient died within 6 hours of admission.

For cases testing positive for EV-71, stratification of the numbers by subgenogroup B5 or C4 is shown.

All patients in the study were admitted to the hospital within the first 8 days of illness (median: 3 days; range: 1–8). EV-A71 and non-EV-A71 EVs were detected in 86% (74/86) of the enrolled patients, with EV-A71 being the most frequently detected (80%, 59/74). EV-A71 was detected in 82% (45/55) of patients presenting with severe disease, including 8/11, 12/14, 21/26 and 4/4 of those presenting with Grade 2b1, 2b2, 3 and 4 disease, respectively. The remaining 10 patients with severe HFMD were either positive for other EVs (n = 5) or PCR negative (Table). While our overall PCR positivity rate was in agreement with previous reports [8], the failure to detect enterovirus in 14% (12/86) of the patients may have been attributed to the low level of viral load in the tested samples and/or the miss-inclusion of patients with other diseases presenting with rash (e.g. chickenpox, measles and rubella) into the study.

**Table:** Demographics, clinical characteristics and aetiology of HFMD patients enrolled in the clinical study, Vietnam, January–September 2018 (n = 86 patients)

Characteristics	Total (n = 86)		EV-A71 (n = 59)		Non-EV-A71 EVs (n = 15)		PCR negative (n = 12)	
Median age in months (range)	21.7 (2.4–60.3)		24.3 (5.4–60.3)		17.6 (2.4–34.6)		15.6 (6.2–33.7)	
Sex ratio, male/female	54/32		36/23		12/3		6/6	
Median illness day on admission (range)	3 (1–8)		3 (2–8)		3 (2–6)		3 (1–7)	
**Origin**	**n**	**%**	**n**	**%**	**n**	**%**	**n**	**%**
Ho Chi Minh City	46	54	33	56	7	47	6	50
Other provinces	40	47	26	44	8	53	6	50
**Clinical grade^a^**	**n**	**%**	**n**	**%**	**n**	**%**	**n**	**%**
2a	31	36	14	24	10	67	7	58
2b1	11	13	8	14	1	7	2	17
2b2	14	16	12	20	2	13	0	0
3	26	30	21	36	2	13	3	25
4	4	5	4	7	0	0	0	0

EV: enterovirus; HFMD: hand, foot and mouth disease.

^a^ According to the Vietnamese Ministry of Health, HFMD is clinically divided into four major Grades, i.e. 1,2,3 and 4 according to increasing disease severity. Grade 2 is further divided in Grade 2a, 2b1, 2b2 [2]. The four Grades are described in more detail in the main text of this report.

Forty partial VP1 sequences were successfully obtained. Sequence analysis revealed that the majority (n = 36, 90%) belonged to EV-A71 subgenogroup C4, while subgenogroup B5 was sporadically detected (n = 4, 10%), (Figure 2). Both rectal and throat swabs of the fatal case were positive for EV-A71 subgenogroup C4.

Of 15 non-EV-A71 enteroviruses, coxsackievirus A6 (CV-A6), CV-A8 and CV-A10 were detected in two, one and eight patients, respectively.

## Discussion and conclusion

HFMD is an emerging infection mostly affecting children ≤ 5 years old [2]. The disease can be caused by various serotypes of the EV**A species, of which EV-A71, CV-A6, CV-A10 and CV-A16 are the most frequently detected serotypes [2,3,9]. In contrast to HFMD associated with other serotypes, the burden of EV-A71 is attributed to both large numbers of hospitalised cases and a relatively high proportion of cases with severe disease and case fatality. While since 2008 (EV-A71-associated) HFMD causes over 1 million reported cases annually across the Asia-Pacific region [10], outbreaks of EV-A71 associated with neurological complications have been documented worldwide, including in Europe and the US [11-13].

An inactivated EV-A71 C4 vaccine has been approved in China in December 2015 but has not been implemented anywhere outside of China [14,15]. Currently, there is no clinically proven effective antiviral drug available, while intravenous immunoglobulin (IVIg) is widely used to treat patients with severe illness [16]. Thus, with poliomyelitis near eradication [17], the emergence of EV-A71 is considered a serious global threat posed by enterovirus infections. Indeed, the World Health Organization has recently included EV-A71 infection for consideration of its Blueprint List of Priority Diseases [18].

EV-A71 exists as a single serotype, but is genetically divided into several genogroups (including A, B and C) and subgenogroups (e.g. B1 to B5 and C1 to C5). While there is no genetic evidence of the difference in virulence between different (sub)genogroups, subgenogroup replacement often accompanies large outbreaks of severe HFMD [19]. Of note, a switch from C5 to C4 in 2011 coincided with an explosive outbreak in Vietnam, which resulted in > 200,000 hospitalisations and > 200 fatal cases between 2011 and 2012 [2]. In contrast, the replacement of C4 by B5 in 2013 coincided with a decrease in the numbers of reported cases and the proportion of cases with severe illness (including fatality) in subsequent years [20,21].

While the underlying mechanism for EV-A71 subgenogroup switches in endemic countries remains unsolved, the observed 2 to 3 year cycles of EV-A71-associated HFMD are attributed to accumulation of a sufficient number of susceptible young children [9].

In Vietnam, HFMD and EV-A71 annual activity increases during the wet season (June to November) and the current upswing of cases is likely to continue over the next few months. Collectively, these data suggest that the re-emergence of subgenogroup C4 after an absence of 6 years causing severe HFMD disease in Vietnam is likely driven by the accumulation of a sufficient number of susceptible young children, and is expected to cause a further increase in monthly hospital admissions. The observed increase of cases, including severe cases, the unprotected birth cohort of children born after 2012 and the known association of EV-A71 C4 with explosive outbreaks and severe illness are therefore a cause of concern of another impending severe outbreak.

Given the absence of vaccines outside China, the absence of specific antiviral treatment and the potential of EV-A71-associated HFMD to spread to other parts of the world including Europe, improving public awareness and public health measures (e.g. hand washing, cleaning/disinfecting surfaces (including toys) and avoiding close contact (hugging and kissing)), as well as accurate case classification and early identification of cases with high risk of deterioration remain essential to minimise the impact of the ongoing outbreak. Clinical trials to demonstrate the effectiveness of current treatment approaches (including IVIg) are of clinical importance and are urgently needed [16].
